# Supplementary material for: Dynamic transcriptomic profiles of zebrafish gills in response to zinc supplementation
Source: BMC Genomics. 2010 Oct 11;11:553. doi: 10.1186/1471-2164-11-553 (PMC3091702; doi:10.1186/1471-2164-11-553)
Supplement: Additional file 2 — Interactive Direct Interaction Network representing the molecular interactions between zinc, copper, iron, calcium and proteins encoded by transcripts changed by zinc supplementation. Mini web-site containing index.html and hyperlinked pages in subdirectory describing a Direct Interaction Network automatically generated based on curated interactions contained within the proprietary PathwayArchitect database. Ovals represent proteins and the circles symbolize metal ions. Objects are coloured by their abundance in zebrafish at the time-point they were significantly different from the control is a scale from -4 fold (dark green) to +4 fold (dark red). Where significant differences were found at more than one time-point, the colour overlay shows expression at the first instance. Dark blue squares denote 'binding', and light blue squares 'expression'; green squares stand for 'regulation', green diamonds for 'metabolism', and green circles for 'promoter binding'. Arrow heads indicate directionality of the interaction where annotated. All nodes and edges can be further interrogated by selecting the relative area of the image. [file 1471-2164-11-553-S2.zip › PathwayArchitect Zn xs DIN/421412.html]

# REGULATION:

|  |  |
| --- | --- |
| Type | REGULATION |
| Effect | None |


---

|  |  |
| --- | --- |
| Score | 0 |


---

|  |  |
| --- | --- |
| Reference Count | 7 |


---

|  |  |
| --- | --- |
| Mechanism | Unknown |


---

|  |  |
| --- | --- |
| Reference:0 || Sentence | "This finding may help to explain what is perhaps the most perplexing question in copper-zinc superoxide dismutase-associated familial ALS-i.e., how such a diverse set of mutations can result in the same gain of function that causes the disease." |
| PMID | 8901564 |
| Year | 1996 |
| Species | Human |
| Journal | Proc Natl Acad Sci U S A |
| RefScore | 1 |
| Source | PArchNLP |
  |
|  |  |


---

|  |  |
| --- | --- |
 Reference:1 || Sentence | "Test cases reported here include the Smoluchowski rate for association of spheres, the association of the enzyme copper-zinc superoxide dismutase with superoxide anion, and the binding of the superpotent sweetener N-(p-cyanophenyl)-N'-(diphenylmethyl)-guanidinium acetic acid to a monoclonal antibody fragment, NC6.8." |
| PMID | 10920003 |
| Year | 2000 |
| Journal | Biophys J |
| RefScore | 1 |
| Source | PArchNLP |
  ||  |  |


---

|  |  |
| --- | --- |
 Reference:2 || Sentence | "The blue crab, Callinectes sapidus, which uses the copper-dependent protein haemocyanin for oxygen transport, lacks the ubiquitous cytosolic copper-dependent enzyme copper/zinc superoxide dismutase (Cu,ZnSOD) as evidenced by undetectable levels of Cu,ZnSOD activity, protein and mRNA in the hepatopancreas (the site of haemocyanin synthesis) and gills." |
| PMID | 12769817 |
| Year | 2003 |
| Species | Human |
| Journal | Biochem J |
| RefScore | 1 |
| Source | PArchNLP |
  ||  |  |


---

|  |  |
| --- | --- |
 Reference:3 || Sentence | "Because copper competes with zinc for intestinal absorption and serum protein binding sites, zinc:copper ratio can reflect the availability of zinc in the body." |
| PMID | 8034078 |
| Year | 1994 |
| Species | Human |
| Journal | Fertil Steril |
| RefScore | 2 |
| Source | PArchNLP |
  ||  |  |


---

|  |  |
| --- | --- |
 Reference:4 || Sentence | Tissue zinc and copper levels did not depict any significant depletion, although changes like marked depletion of zinc following CaNa(2)EDTA and copper after MiADMSA administration were of some concern. |
| PMID | 15452883 |
| Year | 2004 |
| Species | Rat |
| Journal | J Biochem Mol Toxicol |
| RefScore | 3 |
| Source | PArchNLP |
  ||  |  |


---

|  |  |
| --- | --- |
 Reference:5 || Sentence | Copper and zinc were shown to be associated with carbonates and iron-manganese oxides, whereas lead showed the strongest association (72%) with the Fe-Mn oxides. |
| PMID | 15504514 |
| Year | 2004 |
| Journal | Sci Total Environ |
| RefScore | 1 |
| Source | PArchNLP |
  ||  |  |


---

|  |  |
| --- | --- |
 Reference:6 || Sentence | The concentrations and chemical forms of copper (Cu) and zinc (Zn) in surface soils directly influence the movement of Cu and Zn. |
| PMID | 16158580 |
| Year | 2005 |
| Journal | J Environ Sci (China) |
| RefScore | 2 |
| Source | PArchNLP |
  |


---

|  |  |
| --- | --- |
